# Affinity Purification of Binding miRNAs for Messenger RNA Fused with a Common Tag

**DOI:** 10.3390/ijms150814753

**Published:** 2014-08-22

**Authors:** Ke Wei, Feng Yan, Hui Xiao, Xiaoxu Yang, Guie Xie, Ye Xiao, Tingting Wang, Yu Xun, Zhaoqin Huang, Mei Han, Jian Zhang, Shuanglin Xiang

**Affiliations:** 1 Key Laboratory of Protein Chemistry and Developmental Biology of Education Ministry of China, College of Life Science, Hunan Normal University, Changsha 410081, China; E-Mails: weike0831@gmail.com (K.W.); yanfengwujinjin@gmail.com (F.Y.); huix1125@gmail.com (H.X.); yangxiaoxu@hunnu.edu.cn (X.Y.); guiexie@gmail.com (G.X.); xiaoy5488@gmail.com (Y.X.); paopaoguqiqiu@gmail.com (T.W.); xunzeng2@gmail.com (Y.X.); yimijiayou@gmail.com (Z.H.); hanmei@hunnu.edu.cn (M.H.); 2 The Cooperative Innovation Center of Engineering and New Products for Developmental Biology of Hunan Province (20134486), Changsha 410081, China

**Keywords:** affinity purification, mRNA, target miRNA

## Abstract

Prediction of microRNA–mRNA interaction typically relies on bioinformatic methods, but these methods only suggest the possibility of microRNA binding and may miss important interactions as well as falsely predict others. A major obstacle to the miRNA research has been the lack of experimental procedures for the identification of miRNA–mRNA interactions. Recently, a few studies have attempted to explore experimental methods to isolate and identify miRNA targets or miRNAs targeting a single gene. Here, we developed an more convenient experimental approach for the isolation and identification of miRNAs targeting a single gene by applying short biotinylated DNA anti-sense oligonucleotides mix to enhanced green fluorescent protein (EGFP) mRNA which was fused to target gene mRNA. This method does not require a design of different anti-sense oligonucleotides to any mRNA. This is a simple and an efficient method to potentially identify miRNAs targeting specific gene mRNA combined with chip screen.

## 1. Introduction

MicroRNAs (miRNA) are an extensive series of small non-coding RNAs (18–25 nt) with an increasingly recognizable importance in the control of profound regulation of many biological processes in cell proliferation, apoptosis, organogenesis, hematopoiesis, tumorgenesis by regulating gene expression [1]. They are transcribed from intergenic or intronic sequences as long precursors termed pri-miRNAs that are sequentially processed by the enzyme Drosha into ~70 nt pre-miRNAs and Dicer into approximate 22-nt mature miRNAs in the cytoplasm [2,3]. Mature miRNAs regulate gene expression post-transcriptionally by binding principally to the 3'-untranslated regions (3'-UTR) of specific mRNA, thereby guiding the effector proteins of RNAi-induced silencing complex (RISC) into close proximity with the mRNAs [4,5], then the RISC complex will either cleave the target messenger or inhibit its translation [6].

Identification of miRNA–mRNA interaction has been a great challenge. Bioinformatics algorithms have been developed to predict putative miRNA–mRNA interaction [7,8,9]. It has been found that seven to eight nt at the 5' end of the miRNA is the most important determinant for target specificity which called “seed sequence” [10,11]. Many computational approaches for the prediction of miRNA–mRNA interaction are based on complete base pairing to the target mRNA at seed region of miRNA. Although some target genes have been successfully predicted for a few miRNAs, a large number of potential targets are falsely predicted because of perfect seed pairing may not necessarily be a reliable predictor for miRNA interactions [12]. Therefore, biochemical approaches have been designed to address this issue.

**Figure 1:**
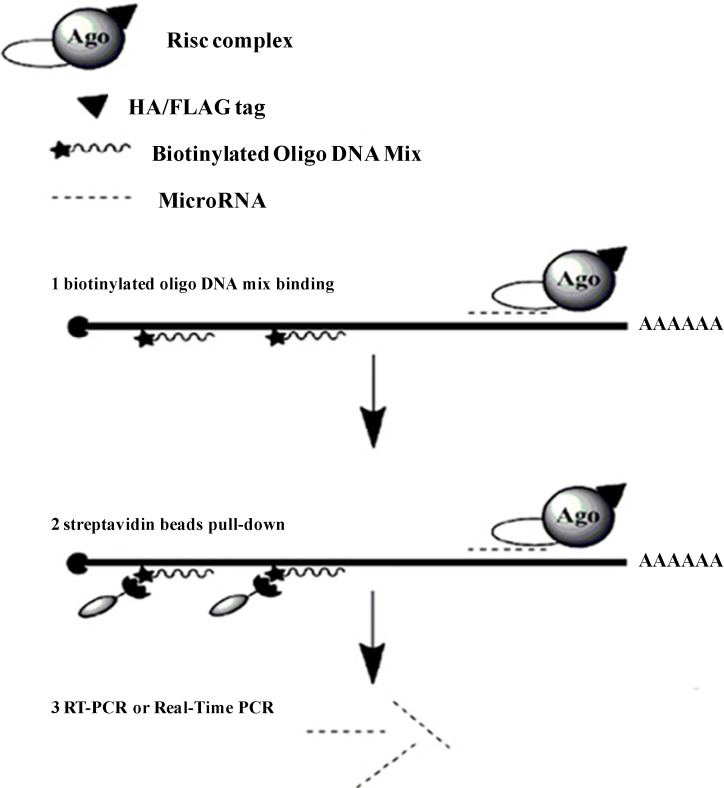
The pull-down strategy of the determination of the microRNAs (miRNAs) that target to a specific gene. Extracts from the cells that over-expressed a tagged Argonaute protein are first incubated with biotinylated oligo DNA mix, and then affinity purified on streptavidin beads. MiRNAs are quantified by real time quantitative PCR (qRT-PCR).

Recently, some biochemical assays are applied to identify new mRNA targets for miRNAs based on affinity purification of labeled miRNA–mRNA complex [13,14,15,16,17]. However, only a few experiment methods have been used to find new miRNAs that regulate one certain gene [18,19]. Here, we have developed a more convenient experimental approach using biotinylated DNA oligonucleotides mix to search for miRNAs that regulate specific mRNA which is fused to enhanced green fluorescent protein (*EGFP*) mRNA (Figure 1), in which the complex of miRNA/mRNA was purified by streptavidin beads through binding to the biotinylated DNA oligonucleotides mix. With the fusion *EGFP* tag, we just need to design biotinylated DNA antisense capture oligonucletides specific to the *EGFP* mRNA instead of designing different antisense oligonucletides for various genes and investigate the transfection efficiency by fluorescence. We selected the programmed cell death protein 4 (*PDCD4*) and miR-21 which are well studied in miRNA–mRNA interaction for this study [20,21,22]. The 3'-UTR of *PDCD4* mRNA contains a MRE (miRNA response element) of miR-21 and was cloned into the downstream of *EGFP*. Using the binding between miRNA and mRNA which was stabilized by formaldehyde and the affinity interaction between biotinylated DNA and streptavidin bead (Thermo Scientific, Waltham, MA, USA), miRNAs targeting the 3'-UTR of *PDCD4* were successfully isolated. As a known target miRNA of *PDCD4*, miR-21 was successfully detected in the isolated miRNAs with polymerase chain reaction (PCR). This experimental approach provides us a simple assay to directly seek out the target miRNAs for any interested gene. Furthermore, this approach is suitable for any miRNAs that target anywhere within the full length of mRNA transcript.

## 2. Results and Discussion

**Figure 2:**
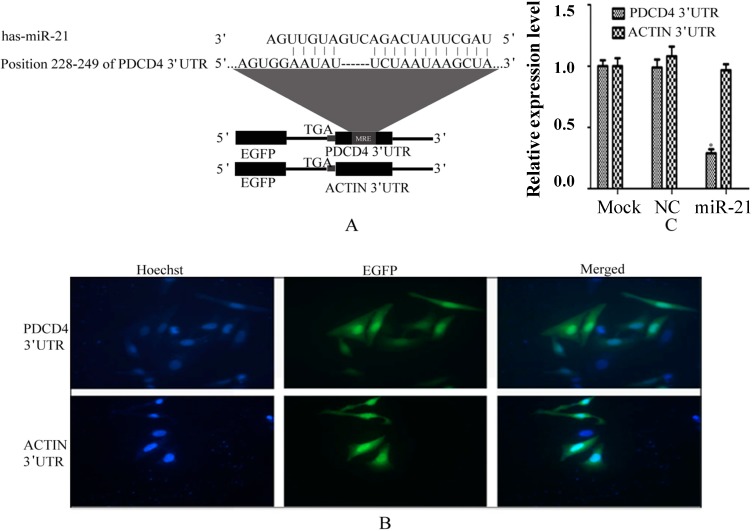
The 3'-untranslated regions (3'-UTR) of programmed cell death protein 4 (*PDCD4*) was regulated by miR-21. (**A**) Diagram of reporter constructs with *EGFP* tag. The 3'-UTR of *PDCD4* with predicted MRE of miR-21 or 3'-UTR of *ACTIN* without MRE were inserted into the downstream of *EGFP* gene of pCMV-enhanced green fluorescent protein (*EGFP*) vector. A stop code was added before the 3'-UTR; (**B**) The expression of *EGFP*-3'-UTR were detected by fluorescence microscope. Nuclear was stained by Hoechst 33258; and (**C**) Repression of *EGFP*-3'-UTR by the interaction between miR-21 and the 3'-UTR. Data are shown as the mean ± SD of three independent experiments.* *p <* 0.05.

**Figure 3:**
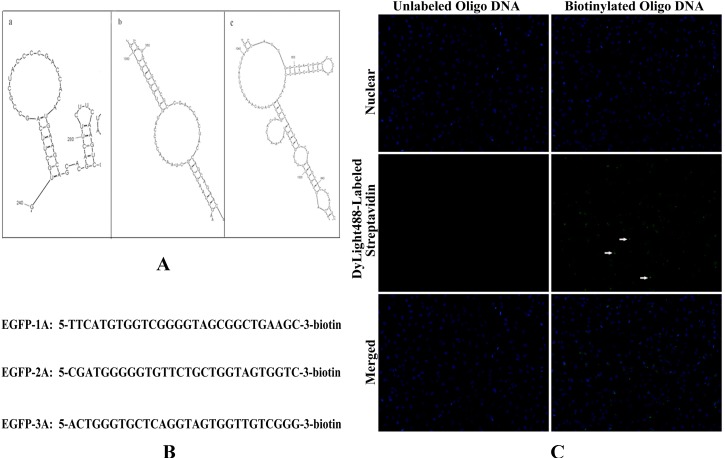
Antisense oligo DNAs of *EGFP* mRNA were labeled with biotin. (**A**) The secondary structures of the *EGFP* mRNA were predicted by software M-Fold; (**B**) The sequence of antisense oligo DNA targeting to *EGFP* mRNA were biotinylated at 3'-end; and (**C**) Biotinylated oligo DNA were detected by fluorescent microscope through Dylight 488-streptavidin.

As reports, *PDCD4* is regulated by miR-21 via its binding to the specific site within 3'-UTR in HeLa cells [20,21]. Therefore, we chose *PDCD4* and miR-21 as a model for this study. About 500 nt 3'-UTR of *PDCD4* which includes miR-21 binding site was inserted into the downstream of *EGFP* (Figure 2A) and 3'-UTR of *β-ACTIN* was also inserted as a negative control which is not regulated by miR-21 [21]. Meanwhile, a stop codon was added between *EGFP* and 3'-UTR. As a result, we can fusion any interested gene into the downstream of *EGFP* that does not influence the *EGFP* expression. Expression of *EGFP-PDCD4* 3'-UTR and *EGFP-ACTIN* 3'-UTR chimeric mRNA were detected by fluorescence microscope. The results show that those chimeric mRNA could be transcripted and translated in HeLa cells (Figure 2B). Then we investigated the binding of miR-21 to the chimeric mRNAs by quantitative real-time PCR using miR-21 mimics transfected with the plasmids above in HeLa cells, and the results showed us that chimeric mRNA of *EGFP-PDCD4* 3'-UTR can be bound by miR-21 (Figure 2C).

The secondary structures of the *EGFP* mRNA were predicted by software M-Fold (mfold.rna.albany.edu/?q=mfold/RNA-Folding-Form). Three exposed single-stranded regions which were present in the first three of the most thermodynamically stringent structures were selected to design DNA oligonucleotide capture sequences (Figure 3A). According to the three predicted secondary structures of *EGFP* mRNA, three 28-nt antisense oligo DNAs targeting to different single-stranded region were designed (Figure 3B). The three antisense oligo DNAs were labeled with biotin at 3'- end and mixed to enhance the binding force to *EGFP* mRNA. To verify the antisense oligo DNAs whether labeled with biotin, we utilized affinity between streptavidin and biotin. Streptavidin conjugated with green fluorescent moiety (Dylight 488-streptavidin) was added into HeLa cells which was transfected with biotinylated oligo DNAs and detected by fluorescence microscope. Compared with unlabeled antisense oligo DNAs, biotinylated oligo DNAs could interact with Dylight 488-streptavidin and show green point distribution in cytoplasm (show as white arrow, Figure 3C). It is demonstrated that antisense oligo DNAs are well biotinylated.

**Figure 4:**
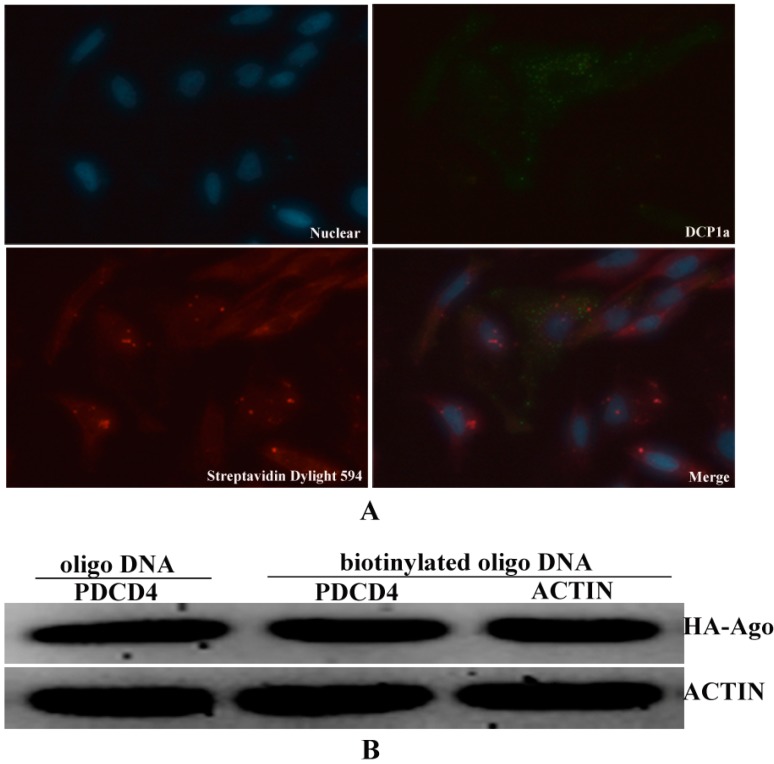
Over-expression of Argonaute 2 protein is to increasing the bingding force between miRNA and mRNA. (**A**) Biotinylated oligo DNA were not localization with P-body in cells; and (**B**) Flag/HA-Ago 2 were detected in the extracts of cells that transfected with Flag/HA-Ago 2 vector.

The biotinylated antisense oligo DNAs showed point distribution in the cytoplasm, so we speculated that the biotinylated oligo DNAs may combine with target *EGFP* mRNA in some complexes that related to the processing and modification of mRNA, such as P-body which was also contributed to the regulation of miRNA and Dcp1a is a marker gene of P-body [23]. To demonstrate the possible interaction between biotinylated oligo DNAs and P-body, the Immunoflurescent assays were performed as described above. The results revealed that biotinylated oligo DNAs and Dcp1a were not co-localized in the cytoplasm (Figure 4A). It is proved that biotinylated oligo DNAs were not localized in P-body. As for this, the binding of biotinylated oligo DNAs to target *EGFP* mRNA may not lead the transcriptional inhibition through the model of P-body [24]. To increase the possible binding of miRNA to target mRNA, we overexpressed AGO2 protein which is a key component of RISC complex that regulated miRNA–mRNA interaction (Figure 4B).

**Figure 5:**
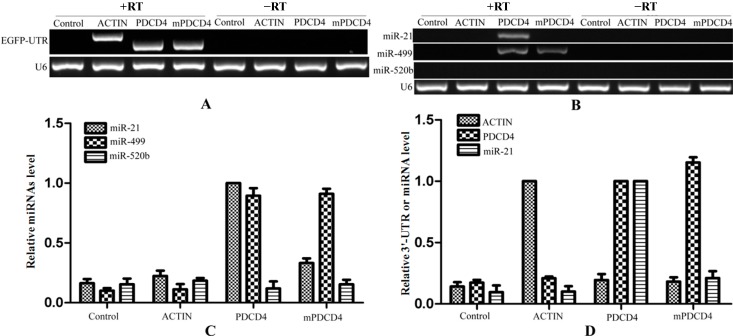
Validation of miRNAs targeting to single mRNA isolation technique. The RNA in the products of pull-down were reverse transcripted and as templates for amplifying. The chimeric mRNAs of *EGFP*-3'-UTR were detected from corresponding products of pull-down (**A**); The enrichment of miRNAs targeting to 3'-UTR-*PDCD4* were shown in the pull-down products using semi-quantitative PCR (**B**) and qRT-PCR (**C**); The 3'-UTRs and miR-21 levels were detected from the pull-down products (**D**). RT: Reverse Transcriptase.

Biotinylated oligo DNAs can combine with target mRNA under the denatured condition and then enriched by streptavidin beads depending on biotin-streptavidin affinity interaction. After 24 h of the transfection of the plasmids in HeLa cells, the complexes of miRNA–mRNA–RBPs (RNA binding proteins) were pulled-down by biotinylated oligo DNAs that bound to target EGFP mRNA. During the process of pull-down, 37% formalin was firstly used for cross-linking among miRNA–mRNA–RBPs which increases the strength of interaction. The cross-link can be reversed under 65 °C for several hours [25]. Hence, the cross-linked protein-RNA complex would not dissociate under 70 °C in a short period of time for unfolding the secondary structures of mRNAs that made the binding of biotinylated oligo DNAs to target *EGFP* mRNA more convenient. Then, we used the streptavidin beads for affinity purification of biotinylated oligo DNAs combined with the miRNA–mRNA–RBPs complexes. Finally, the streptavidin beads were washed three times and the binding RNAs were extracted with Trizol (TaKaRa, Dalian, China) and reverse transcripted using the mRNA and miRNA first strand cDNA synthesis kit independently. During the pull-down process, the 3'-UTR of *PDCD4* that contains a mutated binding site of miR-21 was cloned as a control (mPDCD4). To verify that the target chimeric mRNAs were in the products of pull-down, we amplified the 3'-UTRs of *PDCD4*, *mPDCD4* and *β-ACTIN* using the synthesized cDNA as template (Figure 5A). The forward primers were both designed within the sequence of *EGFP*, while the reverse primers were in the 3'-UTR respectively. Therefore, the products would not be amplified from endogenous *PDCD4* or *β-ACTIN*. As shown in the Figure 5A, *PDCD4*, *mPDCD4* and *β-ACTIN* could be amplified from the templates that reverse transcripted with reverse transcriptase, while not from the templates without reverse transcriptase which excluded the pollution of plasmids transfected. It is demonstrated that the target genes of biotinylated oligo DNAs were pulled-down successfully. Then, we used semi-quantitative PCR and real time quantitative PCR (qRT-PCR) to prove that miR-21 is successfully pulled down followed with the target mRNAs. MicroRNA of *PDCD4* was contained in the products of streptavidin-biotin pull-down. However, the amount of miR-21 was very low, so KOD-FX DNA polymerase (Toyobo, Osaka, Japan) was used to amplify the target miRNA (Figure 5B). MiR-21 was only detected in the product of streptavidin-biotin pull-down from the cells that were transfected with 3'-UTR-PDCD4, while not shown in the cells transfected with 3'-UTR-β-ACTIN or mutated 3'-UTR-PDCD4 (mPDCD4). The product that was pulled down from the cells that were transfected with 3'-UTR-PDCD4 by the oligo DNAs without biotin labeled was also taken as a negative control. All of the templates that were reversely transcribed without reverse transcriptase could not detect the existence of miR-21 (right lines in Figure 5B), and U6 fragments which were added in the elution buffer equally were taken as an endogenous control. The same results were obtained from qRT-PCR (Figure 5C). MiR-21 had a large amount of enrichment only in the product of streptavidin-biotin pull-down from the cells that were transfected with 3'-UTR-PDCD4, while had less in the cells transfected with 3'-UTR-β-ACTIN and the negative control.

MiR-499, as another target of *PDCD4* [26,27], was enriched in the product of streptavidin-biotin pull-down from the cells that were transfected with 3'-UTR-PDCD4 or mutated 3'-UTR-PDCD4, while miR-520b that do not target to 3'-UTR of *PDCD4* was not shown the enrichment (Figure 5B,C). To verify the purification method, qRT-PCR was performed with 3'-UTRs and miR-21 primers. This verification was performed using the 2^−ΔΔ*C*t^ method. As shown in Figure 5D, both the 3'-UTRs of *ß-ACTIN* and *PDCD4* were only enrichment in the product that was transfected with corresponding EGFP-3'-UTR plasmid. The levels of *ß-ACTIN* and *PDCD4* in the product that was transfected with 3'-UTR of *ACTIN* or *PDCD4* were artificially set to 1.0 respectively. The levels of miR-21 had a larger enrichment in the product transfected with 3'-UTR of *PDCD4* (artificially set to 1.0), while less in the product transfected with mutated 3'-UTR of *PDCD4* and other control products. These results demonstrated that our method for isolating the target miRNAs is efficient and specific.

**Figure 6:**
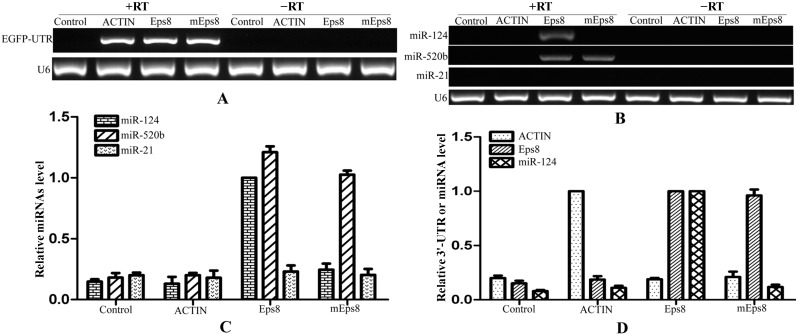
mRNA:miRNAs isolation technique for *Eps8* and its target miRNAs. (**A**) The enrichment of RNA chimeric mRNAs of *EGFP*-3'-UTR demonstrated the efficiency of isolation technique; (**B**) The regulated miRNAs of *Eps8* were obtained from the product that was transfected with 3'-UTR-Eps8 through semi-quantitative PCR. At the same time, miRNAs that without binding sites could not be amplified from these products; (**C**) The levels of detected miRNAs were verified using qRT-PCR after the process of isolation technique; and (**D**) qRT-PCR showing the relationship between mRNA and their target miRNA after the isolation. RT: Reverse Transcriptase.

To validate our affinity purification procedure, another gene and its known regulated miRNAs were processed as described above. It had been reported that *Eps8* could be down-regulated by miR-124 and miR-520b through bind to the 3'-UTR [28]. Over 500 nt 3'-UTR of *Eps8* that contains the binding sites of these two target miRNAs was inserted into the downstream of *EGFP*. Another 3'-UTR of *Eps8* that contains a mutated binding site of miR-124 was also cloned into the downstream of *EGFP* as a control. After pull-down, the three *EGFP*-3'-UTRs were detected from the products that reversely transcripted with reverse transcriptase, while not from the products without reverse transcriptase (Figure 6A). The target miRNAs of *Eps8* were detected using semi-quantified PCR (Figure 6B). MiR-124 and miR-520b could be amplified from the product that transfected with the 3'-UTR of *Eps8*, while miR-124 could not be detected from the product that transfected with mutated 3'-UTR-*Eps8*. The same results were verified using qRT-PCR (Figure 6C). MiR-124 showed a larger enrichment in the product that was transfected with 3'-UTR-*Eps8* (set to 1.0). The mutated 3'-UTR-*Eps8* had an obviously negative effect on the pull-down efficiency of miR-124, while no effect on miR-520b. Meanwhile, miR-21 that do not target to 3'-UTR of *Eps8* had no enrichment in any product (Figure 6B,C). Finally, we also detected the enrichment of UTRs and miR-124 (Figure 6D). These results clearly demonstrated a causal relationship between the enrichment of target miR-124 and the 3'-UTR-*Eps8*, the same as miR-21 and 3'-UTR-*PDCD4* (Figure 5D). It is suggested that our experimental approach can isolate the target miRNAs for a single gene successfully.

## 3. Experimental Methods

### 3.1. Cell Culture and Transfection

Human cervical cancer HeLa cells were cultured in Dulbecco’s modified Eagle’s medium (DMEM, Thermo, Waltham, MA, USA) containing 10% fetal bovine serum (Thermo, Waltham, MA, USA), 100 units/mL penicillin (Beyotim, Beijing, China), 100 µg/mL streptomycin (Beyotim, Beijing, China) and 2 mM l-glutamine (Invitrogen, Carlsbad, CA, USA) at 37 °C and 5% CO_2_. Antisense strand DNAs labeled with biotin were synthesized by Genepharma. Expression vectors described in this study were transfected using Lipofectamine 2000 (Invitrogen, Carlsbad, CA, USA) according to the manufacturer’s instructions.

### 3.2. Plasmid Constructs

The 3'-UTR of *PDCD4*, *Eps8* and *β-ACTIN* were amplified from HeLa cDNA and then inserted into the 3'-end of the *EGFP* gene of the expression vector pEGFP-C1 (Clontech, Palo Alto, CA, USA) between BglII and EcoRI sites. The primers used were: *PDCD4* 3'-UTR-F (5'-AGGAGATCTTTTTGAGTACAAGGCATTTCTGAC-3') and *PDCD4* 3'-UTR-R (5'-AAGGAATTCAGATGTTCCAGCCACCTTTTACTT-3'), *Eps8* 3'-UTR-F (5'-ATGAGATCTCTATAGAGCATTCTCAAGAC-3') and *Eps8* 3'-UTR-R (5'-AGGGAATTCCATTTATTAACAGACAGCAA-3'), *β-ACTIN* 3'-UTR-F (5'-TTAAGATCTCCATAGTCCACCGCAAAT-3') and *β-ACTIN* 3'-UTR-R (5'-ATAGAATTCCTGCCTCCACCCACTCCC-3'). Both of the forward primers were added a stop code TAG at the 3'-flanking of restriction sites. Translation of 3'-UTR was prevented by the stop code, but ineffective to the mRNA of *EGFP*. The binding site of miRNA during 3'-UTR of *PDCD4* or *Eps8* was deleted mutated and cloned into downstream of *EGFP* according to the references [20,28]. Finally, plasmids were sequenced and characterized.

### 3.3. Predicted the Secondary Structure of EGFP by Bioinformatics and Designed the Antisense Strand DNA of EGFP

*EGFP* mRNA could be used as an RNA tag and detect the transfection efficiency through fluorescence microscope. The secondary structure of *EGFP* mRNA was predicted by mfold (http://mfold.bioinfo.rpi.edu/cgi-bin/ rna-form1.cgi) and then we designed the antisense strand DNA using Primer premier 5.0 (Primer Biosoft, Palo Alto, CA, USA). The sequence of antisense strand DNA was designed according to the first three predicted secondary structure. Three antisense strand DNAs were designed at different parts of the ring structure and labeled with biotin at the 3'-end by Sangon Biotech (Shanghai, China). All of the three biotinylated DNAs were mixed used to enhance the ability of pull-down during this assay.

### 3.4. Western Blot

HeLa cells were washed twice with ice-cold PBS and lysed in lysis buffer (1 M Tris–HCl, pH 7.5, 4 M NaCl, 1% tristonx-100, 10 mg/mL deoxycholate sodium, 1 mg/mL Sodium dodecyl sulfate, 1% cocktail). 20 μg of soluble protein was separated by SDS-PAGE and transferred to PVDF membranes (Millipore, Darmstadt, Germany). Primary antibody against flag (Sigma, St. Louis, MO, USA) was used in this report. The protein was detected using a HRP-conjugated secondary antibody and SuperSignal West Pico Chemiluminescent substrate kits (Pierce, Wayland, MA, USA).

### 3.5. Immunocytochemical Staining

HeLa cells were grown on sterilized coverslips until 60%–80% confluent and then transfected using Lipofectamine2000 (Invitrogen, Carlsbad, CA, USA) for 24 h. Cells were washed twice with ice-cold PBS and then fixed with 4% (*w*/*v*) paraformaldehyde in PBS for 10 min, and permeabilized with acetone and methanol (1:1) for 30 s. Cells were then incubated with primary antibodies diluted in PBS with 10% (*v*/*v*) normal goat serum for 1 h and with the secondary antibody under the same conditions. The primary antibody was anti-streptavidin (Thermo, Waltham, MA, USA) that marked by DyLight 488 (Molecular Probes, KPL, Gaithersburg, MD, USA). Hoechst 33258 (Sigma, St. Louis, MO, USA) was used to stain the nuclei. Fluorescence on the processed slips was analyzed using a fluorescence microscope (Zeiss, Oberkochen, Germany).

### 3.6. Streptavidin-Biotin Pull-Down

HeLa cells were transfected using Lipofectamine 2000 (Invitrogen, Carlsbad, CA, USA) for 24 h. 37% formalin was added for 10 min at room temperature and then additional 1 mL 10× Glycine for 5 min to stop the cross-linking reaction. The cells were washed twice with ice-cold PBS and collected into a new RNase-Free tube. HeLa cells were lysed with RIPA buffer (50 mM Tris–HCl (pH 7.2), 150 mM NaCl, 1% (*v*/*v*) Triton X-100, 1% (*w*/*v*) sodium deoxycholate, 0.1% (*w*/*v*) SDS) in ice-water for 30 min. Centrifuged and the supernatant was denatured in 70 °C for 5 min to depolymerize the secondary structure. Supernatant was mixed with biotinylated DNA of EGFP for 1 h at room temperature and then incubated with equilibriumed streptavidin beads. Streptavidin beads were washed 4 times with 10% glycerol, 20 mM Tris (pH 8.0), 0.2 mM EDTA, 0.5% NP-40, 0.35 M KCl, 1 mM DTT. Finally, the complex of biotin-DNA:mRNA:miRNA with the magnetic beads were collected by centrifugation.

### 3.7. qRT-PCR

For qRT-PCR, RNAs in the complex of biotin-DNA:mRNA:miRNA were directly extracted using Trizol (Invitrogen, Carlsbad, CA, USA) after being incubated with Proteinase K (Sigma, St. Louis, MO, USA) in 55 °C for 10 min. The extracted RNAs were reverse transcripted with commercially available NCode™ miRNA First-Strand cDNA Synthesis and qRT-PCR assay (Promega, Madison, WI, USA). The extracted RNAs were also reverse transcripted with Reverse Transcription System (Promega, Madison, WI, USA) and used for the amplifying 3'-UTRs of *PDCD4* and *β-ACTIN.* The resulting cDNA was amplified using oligonucleotide primers and SYBR Green MasterMix (TaKaRa, Dalian, China) for qRT-PCR. Relative copies of miR-21 were determined using the 2^−ΔΔ*C*t^ method and the U6 fragments served as an endogenous control. Oligonucleotide primers used were as follows: *EGFP* forward, 5-GCAGAAGAACGGCATCAAG-3; *PDCD4* reverse, 5-ATGTTCCAGCCACCTTTTACTT-3; *ACTIN* reverse, 5-CTGCCTCCACCCACTCCC-3; *Eps8* reverse, 5-TTAGAGAAATTATCTAGATTC-3. The forward qRT-PCR primers of the miRNAs were purchased from GeneCopoeia (Guangzhou, China). The reverse qRT-PCR primers of the miRNAs were used NCode™ miRNA First-Strand cDNA Synthesis and qRT-PCR kit (Invitrogen, Carlsbad, CA, USA).

### 3.8. Statical Analysis

GraphPad Prism 5 was used for statistical analysis. All results were presented as mean ± SD. The significance of differences between samples were performed by Student’s *t*-test and defined as *p < 0.05.

## 4. Conclusions

In conclusion, these results showed us that our affinity purification procedure for gene-specific targeting microRNAs is effective, specific, and provide us a widely applicable method for any interest gene avoiding a design of biotinylated oligo DNAs to any interested gene. The use of *EGFP* mRNA as a tag mRNA to design biotinylated antisense oligo DNAs is sufficient and the EGFP can also indicate transfection efficiency. However, considering that EGFP plasmid is about 4 kb, the 3'-UTR length of interested gene cannot be too long to lead to low transfection efficiency. Therefore, the necessary fragment of 3'-UTR is needed to decrease the length of fragment inserted following the *EGFP*. This method is not based on bioinformatics prediction and can purify the target miRNAs through the experimental method. Furthermore, our procedure might discover unpredicted target microRNAs when combined with High throughput miRNA sequencing and, at least, lead to a significantly advance in miRNA research.
